# Advances in Targeted Therapy: Addressing Resistance to BTK Inhibition in B-Cell Lymphoid Malignancies

**DOI:** 10.3390/cancers16203434

**Published:** 2024-10-10

**Authors:** Andres Bravo-Gonzalez, Maryam Alasfour, Deborah Soong, Jose Noy, Georgios Pongas

**Affiliations:** 1 London School of Hygiene and Tropical Medicine, Bogotá 110221, Colombia; lonab41@student.london.ac.uk; 2 Department of Medicine, University of Miami and Jackson Memorial Hospital, Miami, FL 33136, USA; mxa1900@miami.edu (M.A.); dxs842@med.miami.edu (D.S.); jin18@med.miami.edu (J.N.); 3 Division of Hematology, Department of Medicine, University of Miami and Sylvester Comprehensive Cancer Center, Miami, FL 33136, USA

**Keywords:** lymphoma, B-cell, hematologic neoplasms, Bruton’s tyrosine kinase, protein kinase inhibitors, drug resistance, neoplasm, ibrutinib, acalabrutinib, zanubrutinib, pirtobrutinib, molecular targeted therapy, chronic lymphocytic leukemia, hematopoietic stem cell transplantation, covalent inhibitors, protein degradation

## Abstract

**Simple Summary:**

Bruton’s tyrosine kinase inhibitors (BTKi) have revolutionized the treatment of various B-cell lymphoid malignancies. However, acquisition of resistance to BTKi has emerged as an important clinical challenge. Herein, we provide a detailed review, describing the clinical trials that led to the FDA approval of the BTK inhibitors for management of Chronic Lymphocytic Leukemia (CLL), Mantle Cell Lymphoma, Marginal Zone Lymphoma (MZL), Follicular Lymphoma (FL) and Waldenstrom Macroglobulinemia (WM). We describe the mechanism of intrinsic and extrinsic resistance to the BTKi with main emphasis on the functional description of BTK mutations in CLL. Finally, we review the latest updates of the PROTACs BTK degraders, an evolving treatment modality for the management of the BTKi refractory B-cell malignancies.

**Abstract:**

B-cell lymphoid malignancies are a heterogeneous group of hematologic cancers, where Bruton’s tyrosine kinase (BTK) inhibitors have received FDA approval for several subtypes. The first-in-class covalent BTK inhibitor, Ibrutinib, binds to the C481 amino acid residue to block the BTK enzyme and prevent the downstream signaling. Resistance to covalent BTK inhibitors (BTKi) can occur through mutations at the BTK binding site (C481S) but also other BTK sites and the phospholipase C gamma 2 (PLCγ2) resulting in downstream signaling. To bypass the C481S mutation, non-covalent BTKi, such as Pirtobrutinib, were developed and are active against both wild-type and the C481S mutation. In this review, we discuss the molecular and genetic mechanisms which contribute to acquisition of resistance to covalent and non-covalent BTKi. In addition, we discuss the new emerging class of BTK degraders, which utilize the evolution of proteolysis-targeting chimeras (PROTACs) to degrade the BTK protein and constitute an important avenue of overcoming resistance. The moving landscape of resistance to BTKi and the development of new therapeutic strategies highlight the ongoing advances being made towards the pursuit of a cure for B-cell lymphoid malignancies.

## 1. Introduction

Non-Hodgkin’s lymphomas are a diverse group of lymphoid malignancies that represent the fourth most common cancer and the sixth leading cause of cancer death in the United States [1]. Treatment has traditionally required the use of chemotherapy, which caused a wide variety of adverse events, but the paradigm has changed since the introduction of targeted therapies. Bruton’s tyrosine kinase (BTK), a key enzyme of the TEC family of kinases, is crucial for the normal function and development of B-cells. Because of the reliance of B-lymphocytes to the BTK, its inhibition also constitutes an important approach in the treatment of B-cell malignancies including the chronic lymphocytic leukemia/small lymphocytic lymphoma (CLL/SLL), mantle cell lymphoma (MCL), Waldenström’s macroglobulinemia (WM), Marginal Zone lymphoma (MZL) and Follicular lymphoma (FL) [2].

The introduction of covalent BTKi marked a significant shift in treating these conditions. Ibrutinib, the first of these inhibitors, targets the BTK enzyme by binding to the C481 residue site, blocking the ATP-binding pocket and therefore inhibiting its catalytic activity [3]. This interaction prevents BTK from participating in the B-cell receptor signaling process downstream of the B-cell receptor (BCR). Early clinical trials of Ibrutinib demonstrated evidence of resistance to the covalent BTKi, which primarily occurred through mutations at the BTK C481 binding site and the PLCγ2, a downstream substrate of BTK [4].

To overcome these resistance mechanisms, noncovalent (reversible) BTKi were subsequently developed. These newer agents do not rely on binding to the C481 residue and can inhibit both wild-type and the C481S mutant, resulting in favorable clinical outcomes as shown in the BRUIN Phase 1–2 trial [5,6]. Herein, we describe the genetic and molecular mechanisms of acquisition of resistance to covalent and non-covalent BTKi. Additionally, we present a new category of emerging BTK directed therapies, such as the BTK degraders, which result in proteolysis of the BTK protein, offering another novel mechanism to overcome resistance to BTKi [7].

## 2. Bruton Tyrosine Kinase Protein

### 2.1. History of BTK Discovery

BTK enzyme is a non-receptor member of the TEC family of non-receptor tyrosine kinases. BTK is mutated in the X-linked agammaglobulinemia (XLA), a rare genetic disorder which was initially described in 1952 [8]. In 1993, two groups identified that the BTK protein is mutated in patients with XLA, leading to compromised B-cell development in the bone marrow with subsequent depletion of serum immunoglobulins and humoral immunodeficiency [9,10]. This was further confirmed by Khan et al., showing that introduction of specific mutations to BTK in mouse models, can lead to diminished BTK expression, resulting in reduced numbers of mature conventional B-cells and IgM and IgG3 deficiency [11].

### 2.2. Molecular Family, Biochemistry and Activation of BTK

The TEC family is the second largest non-receptor tyrosine kinase family and is comprised of five proteins: the bone-marrow expressed kinase (BMX), the interleukin-2-inducible T-cell kinase (ITK), the tyrosine kinase expressed in hepatocellular carcinoma (TEC), the tyrosine kinase protein (TXK) and the BTK [2]. The BTK protein has five main domains: (i) The N-terminal pleckstrin domain, (ii) a TEC homology domain, (iii/iv) two SRC homology domains (SH3 followed by SH2) and (v) the kinase domain, which harbors the enzymatic activity and transfers the phosphate group from ATP to tyrosine residues on substrate proteins [12]. BTK becomes active after cell membrane association and subsequent phosphorylation of the Y551 in the kinase domain by the upstream proteins of the SRC family kinase or by the spleen tyrosine kinase (SYK) [13]. These events activate the catalytic activity of BTK and result in autophosphorylation of Y223 in the SH3 domain [14]. While phosphorylated Y223 has shown to mirror the catalytic activity of the BTK, it is unclear whether it influences the biological function of BTK [15].

### 2.3. BTK in the B-Cell Receptor Signaling Pathway

**Figure 1:**
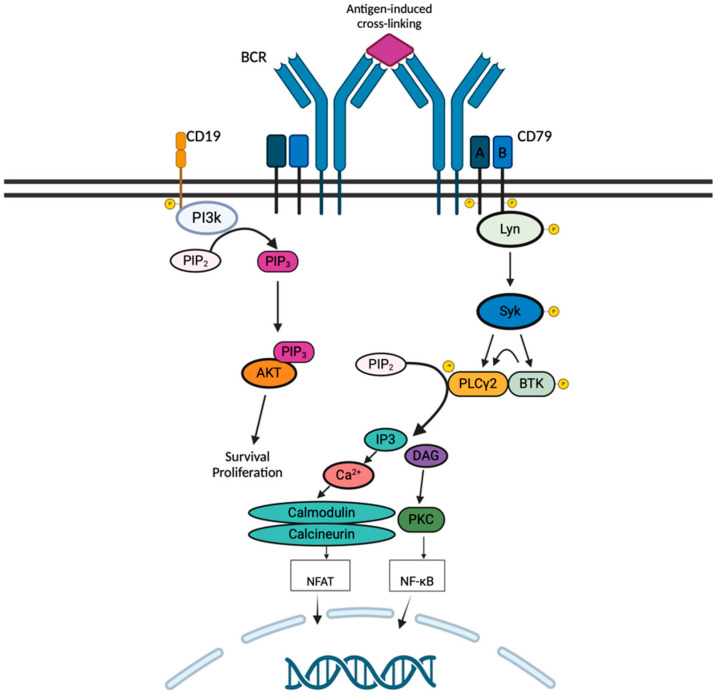
BCR signaling in a B-cell lymphocyte.

The IgM B-cell receptor pathway (BCR) is essential for the survival of the peripheral B-lymphocytes (Figure 1) [16]. Because of its short cytoplasmic domain, the IgM cannot signal directly but it associates with the transmembrane proteins CD79a/CD79b. This heterodimer contains the immunoreceptor tyrosine-based activation motifs (ITAMs) in their cytoplasmic domain, which can be phosphorylated by the Src-family protein tyrosine kinases, such as LYN, upon BCR engagement by an antigen, with subsequent creation of docking sites for the spleen tyrosine kinase (SYK) [17]. Upon BTK activation by SYK, BTK leads to phosphorylation of PLCγ2 at Y753 and Y759 which are important for its catalytic activity [18]. The active PLCγ2 cleaves the phosphatidylinositol bisphosphate (PIP2) into two second messengers: diacylglycerol (DAG) and inositol triphosphate (IP3). The IP3 regulates the intracellular calcium and leads to activation of the activated T cells (NFAT) transcription, through calcineurin and calmodulin. The DAG activates protein kinase Cβ (PKCβ), which oversees the activation of IκB kinase (IKK) proteins and the downstream NF-κB pathway. In summary, BTK links the BCR pathway to the activation of the NF-κB, a vital pathway for B-cell proliferation, maturation, and differentiation [19].

### 2.4. The Role of BTK in B-Cell Malignancies

The central role of BTK in B-cell malignancies was first discovered by Davis et al., through RNA interference genetic screen in human lymphoma cell lines, demonstrating that BTK is essential for the survival of the activated B-cell-like (ABC) a subtype of diffuse large B-cell lymphoma (DLBCL) addicted to the NF-κΒ pathway [20]. These experiments were also recapitulated through pharmacological inhibition of BTK with the Ibrutinib (formerly known as PCI-32765), a first in class BTK inhibitor. Ibrutinib forms an irreversible covalent bond with the cysteine residue on C481, resulting in the inhibition of the BTK enzymatic activity, abrogation of the autophosphorylation at Y223 and subsequent halting of the downstream BCR signaling [3,21]. Besides the ABC-DLBCL, Ibrutinib demonstrated activity in preclinical models of CLL, where it was shown to abrogate downstream survival pathways including ERK1/2, PI3K and NF-κB, providing solid and significant support for the development of Ibrutinib as a therapeutic agent across a variety of B-cell malignancies [22].

### 2.5. Covalent BTK Inhibitors in Clinical Practice

#### 2.5.1. Ibrutinib: First-Generation Covalent BTK Inhibitor

The first clinical data demonstrating the fundamental therapeutic role of BTKi in patients with relapsed/refractory B-cell lymphoma and CLL were described in a phase I trial by Advani et al., where Ibrutinib (Imbruvica) showed an objective response rate (ORR) of 60% (Table 1) [23]. The first FDA approval of Ibrutinib was obtained in 2013 for the treatment of relapsed/refractory (R/R) mantle cell lymphoma (MCL) based on a phase II trial (PCYC-1104), which showed an ORR of 67%, a complete response (CR) of 23%, median progression-free survival (mPFS) of 13 months and median overall survival (OS) of 22.5 months [24,25]. In 2014, Ibrutinib was approved for patients with R/R CLL, who had received at least one prior therapy, based on the RESONATE study, a multicenter open-label phase 3 study, where Ibrutinib was compared to Ofatumumab, an anti-CD20 monoclonal antibody. The trial was stopped early for efficacy after demonstrating 78% reduction in risk of progression or death. Ibrutinib demonstrated an ORR of 42.6% vs. 4% with Ofatumumab and a mPFS of 44.1 months vs. 8.1 months [26,27]. Subsequently in 2015, ibrutinib became the first FDA approved therapy for Waldenström’s Macroglobulinemia (WM), based on phase II study showing an ORR of 90.5% and a major response rate of 73% [28]. Following the FDA approvals of Ibrutinib in the R/R setting for MCL, CLL and WM, subsequent studies demonstrated benefit in the front line, gaining new indications for treatment-naïve patients with CLL and WM [29,30]. Ibrutinib was also investigated in R/R DLBCL in a phase 1/2 study, where it demonstrated objective responses in 39% of patients with ABC-DLBCL, albeit with short mPFS of 2 months [31]. Those results prompted a randomized phase III trial evaluating Ibrutinib plus R-CHOP (rituximab, cyclophosphamide, doxorubicin, vincristine, and prednisone) vs. placebo plus R-CHOP in treatment-naive non-germinal center B-cell DLBCL (non-GCB DLBCL) [32]. While this study did not meet the primary end point of event free survival (EFS) in the intent to treat (ITT) and the ABC-DLBCL subgroup, patients below 60-years of age demonstrated improved EFS, PFS and OS, but with some increased adverse effects. 

Notably, a common observation among the clinical trials and real-world experience of Ibrutinib in lymphoid malignancies, was the high rates of cardiovascular adverse events including hypertension, atrial fibrillation and bleeding. Because of the increased toxicity of the ibrutinib in the confirmatory trial of SHINE and not meeting the primary end point of PFS in the SELENE study, Abbvie withdrew the ibrutinib indications for patients with MCL who have previously received at least 1 therapy and marginal zone lymphoma (MZL) for patients who required systemic therapy and had received at least 1 prior anti-CD20-based therapy.

#### 2.5.2. Second Generation Covalent BTK Inhibitors: Acalabrutinib and Zanubrutinib

Acalabrutinib (Calquence) was the first of the two second-generation covalent-BTKi to obtain FDA approval. In 2017, the phase study ACE-LY-004 enrolled patients with R/R MCL and demonstrated an ORR of 81%, with 48% CR and a median PFS of 22 months [34,47]. Subsequently in 2019, Acalabrutinib was approved for adults with CLL based on the ELEVATE-TN and the ASCEND trial for treatment-naive patients (TN) and patients with R/R disease, respectively. The ELEVATE-TN compared Acalabrutinib alone or in combination with Obinutuzumab vs. Chlorambucil plus Obinutuzumab, and demonstrated a superior mPFS at a median follow-up of 28.3 months (mPFS not reached), compared with 22.6 months for Obinutuzumab-Chlorambucil [35]. The ASCEND trial compared Acalabrutinib to Idelalisib or Bendamustine plus Rituximab, and after a median follow-up of 16.1 months, the mPFS was significantly longer for the Acalabrutinib (not reached) compared to the investigator’s choice of 16.5 months [39].

Zanubrutinib (Brukinsa) the newest of the 2nd generation BTKi, received the first approval in 2019 for R/R MCL based on two single arm clinical trials which assessed ORR as primary endpoint and collectively demonstrated an ORR of 84% [40,41]. In 2021, Zanubrutinib was granted its second approval for the treatment of WM based on ASPEN trial, a phase 3 study, comparing Zanubrutinib with Ibrutinib in patients with WM [48]. In the first cohort of the MYD88 with L265P mutation, the CR and very good partial response (VGPR) were 36.3% for Zanubrutinib vs. 25.3% for Ibrutinib, whereas in the second cohort with the MYD88 wildtype or unknown mutational status, the response rates were 30.8% with one CR [42]. Subsequently, the approval for R/R MZL was granted in 2021 based on the efficacy results from MAGNOLIA and the BGB-3111-AU-003 trial [49]. The MAGNOLIA phase 2 study demonstrated an ORR of 68.2% with a CR of 25.8%, whereas the BGB-3111-AU-003, a phase 1/2 study of Zanubrutinib in B-cell malignancies showed an ORR of 80.0% with a CR of 20% in MZL [41,43]. Regarding the approval of Zanubrutinib in CLL, it was granted based on two phase 3 randomized trials. The SEQUOIA study compared Zanubrutinib versus Bendamustine and Rituximab in TN-CLL and met the prespecified criteria for superiority by 58% improvement of the PFS [44]. In the R/R setting, the ALPINE trial compared Zanubrutinib vs. Ibrutinib and demonstrated a higher ORR 83.5% vs. 74.2% and a 35% improvement of PFS [45]. The most recent approval of Zanubrutinib was granted in 2024 for follicular lymphoma based on the ROSEWOOD trial, which compared Zanubrutinib plus Obinutuzumab vs. single agent Obinutuzumab. The study showed an ORR of 69% for the combination vs. 46% for the Obinutuzumab monotherapy with a 18-month duration of response (DoR) of 69% [46].

Collectively, these clinical data highlight the importance of the covalent BTKi in the treatment of various B-cell malignancies, but even more importantly underscore the fact that the duration of response can be limited due to acquisition of resistance.

### 2.6. Resistance Mechanisms to Covalent BTK Inhibitors

Despite the remarkable efficiency of the covalent BTKi in the treatment of B-cell malignancies, resistance can develop. From a clinical standpoint, resistance is classified as primary or secondary. Primary resistance is defined as resistance that occurs in patients who fail to respond to the BTKi upfront, whereas secondary resistance is one that develops in patients who initially responded to the BTKi and then developed relapse. From a biological standpoint, the mechanisms of resistance can be broken down to 1. Intrinsic mechanisms involving mutations of the cancer cells and upregulation of survival signaling pathways and 2. Extrinsic mechanisms involving the tumor microenvironment. Herein, we primarily focus on the secondary mechanisms of resistance which are more frequently observed in the clinical setting of CLL with more emphasis on the acquisition of BTK mutations.

For CLL patients treated with ibrutinib, those with primary refractory disease or early progression within 15 months for the initiation of therapy often present with Richter’s syndrome, a histologic transformation of CLL to DLBCL [50]. In contrast, progression due to CLL occurs later with acquisition of mutations to BTK C481S and PLCγ2 (within the autoinhibitory domain) in approximately 80% of patients with late progression [51].

**Figure 2:**
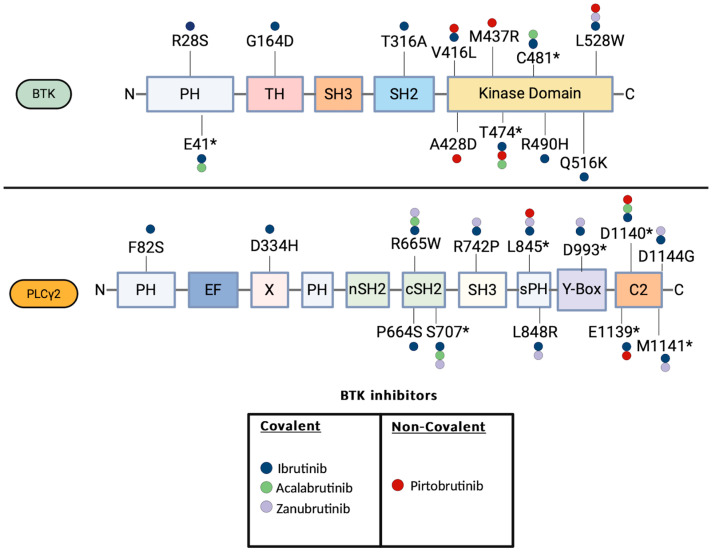
BTK and PLCγ2 mutations described in patients treated with the FDA-approved BTKi Ibrutinib, Acalabrutinib, Zanubrutinib and Pirtobrutinib. The domains of the BTK and PLCγ2 are illustrated. BTK E41*: E41 K/V, BTK C481*: C481 S/F/T/Y/R/G, BTK T474*: T474I/F. PLCγ2 S707*: S707 Y/F/P, L845*: L845 F/L, D993*: D993 G/H/Y, D1139*: D1139G/del, D1140*: D1140 E/G/N, M1141*: M1141 K/R.

While the BTK C481S is the most commonly observed mutation described in all the three FDA-approved covalent BTKi, mutations to other amino acid residues have been described as well (Figure 2) [52,53,54,55]. With functional studies based on enzymatic activity and a differential interactome, two distinct classes of BTK mutations of the kinase domain have been defined including the Kinase Proficient drug resistance mutations (C481S and T474I) and the Kinase Deficient/Dead drug resistance mutations (L528W) [7]. Similarly to the C481S mutation, the T474I mutation can increase the autophosphorylation of BTK-Y223 in the absence of BCR stimulation and activate the downstream signaling. While T474I has been described both in R/R CLL cases on Ibrutinib and Acalabrutinib, it has not yet been described in Zanubrutinib, highlighting that the acquisition of resistance to the covalent BTKi does not have a universal mechanism that can be applied for every BKTi, but there can be specific mechanisms for each individual BTKi [56,57].

Regarding the Kinase Deficient/Dead drug resistance mutations, the L528W mutation has been described in patients with Ibrutinib and Zanubrutinib [53,58]. L528W mutation is associated with reduced autophosphorylation of the BTK-Y223 and reduced BTK enzymatic activity. Functional experiments with immunoprecipitation mass spectrometry have revealed a scaffold function for the BKT L528W with downstream cell signaling, which skews the survival dependence on surrogate kinases that can bind to kinase-impaired BTK proteofolds.

In an attempt to characterize the clonal evolution and frequency of BTK mutations for CLL patients progressing on ibrutinib or acalabrutinib, Woyach et al. identified that BTK mutations occur in 66% of acalabrutinib- and 37% of ibrutinib-treated patients [59]. The BTK C481S mutation was the most common mutation in both groups, among those who acquired BTK mutations, occurring in 93.5% of acalabrutinib- and 90.9% of ibrutinib-treated, followed by the T474I mutation in 29% of the acalabrutinib- but none of the ibrutinib-treated population.

Directly downstream of the BTK, mutations in PLCγ2 can also lead to resistance. Several nonsynonymous mutations in PLCγ2 have been described in Ibrutinib-resistant CLL, with the majority of them occurring in the SH2 domain including the P664S, R665W and S707* [51,55,56,59,60,61,62,63]. Notably, mutations of the PCLγ2 SH2 domain have been shown to activate the PCLγ2 independently of the BTK, and reconstitute the BCR signaling, which can explain their acquisition in the context BTK inhibition [64,65]. Besides the mutations of BTK and PCLγ2, studies with whole exome and deep-targeted sequencing studies have also described the clonal evolution of CLL developing resistance to BTK inhibition with expansion of clones with deletion of 8p and additional driver mutations of EP300, MLL2, EIF2A [61,66].

In MCL, in contrast to the CLL, primary resistance to Ibrutinib is more common, occurring in 32%, which could stem from the fact that MCL has a higher rate of high-risk mutations such as mutations in the ATM and TP53 gene in 44% and 27%, respectively [24] [67]. Primary resistance is mainly mediated though genetic lesions in the alternative NF-κΒ pathway as shown in human cell lines and MCL samples with mutations in TRAF2 and BIRC3 [68] and also, through activation of compensatory signaling pathways such as the PI3K/AKT/mTOR, MEK/ERK and canonical NF-κB pathway. Unlike CLL, mutations in the BTK are rarely detected at disease progression. Similarly to primary resistance, secondary resistance is mainly mediated through the alternative compensatory signaling pathways, albeit mutations have been described in D1 (CCND1) gene and CDKN2A/MTAP gene [69].

In ABC-DLBCL that appear to be addicted to the BTK, as shown by high throughput experiments with siRNAs and sgRNA, the short PFS of 2 months achieved by ibrutinib in ABC-DLBCL in the 1/2 study pointed towards rapid acquisition of resistance. Through experimental modeling of resistance of ABC-DLBCL cell lines to ibrutinib, it was demonstrated that the primary mode of resistance is primary epigenetic and partially driven by the transcription factor TCF4. Specifically, the TCF4 expression led to substitution of the BTK for RAC2 in the activation of the PLCγ2 and downstream activation of the NF-κB pathway [70].

Besides the aforementioned intrinsic mechanisms of resistance, extrinsic alterations in the tumor microenvironment have also been implicated in the acquisition of resistance. Through activity-based protein profiling (ABPP) and cell-based drug screening in MCL cell lines followed by validation in ibrutinib-resistant MCL human samples, it was demonstrated that ibrutinib-resistant MCL cells are characterized by activation of the PI3K-AKT-motR pathway and overexpression of integrin-β1, which can facilitate adhesion to stroma cells and clonogenic growth in the presence of ibrutinib [71]. Furthermore, micro-environmental agonists such as interleukin-10, CD40 ligand, and cytosine guanine dinucleotide–oligodeoxynucleotides (CpG-ODNs) when incubated with MCL and CLL cells can lead to antiapoptotic protein expression through activation of the NF-κB pathway [72].

### 2.7. Noncovalent BTK Inhibitors: Clinical Data and Emergence of Resistance

The acquisition of resistance to the covalent BTKi, poses a major challenge in the management of patients with CLL or NHL. Noncovalent or reversible BTKi were originally developed to bind and inhibit the wild type and mutated C481S BTK [60]. Preclinical studies assessing Pirtobrutinib (LOXO-305), demonstrated inhibition of the BCR pathway in the presence of the C481S mutation, which moved this agent to the clinic [73].

The efficacy of Pirtobrutinib was assessed in the BRUIN study, an open-label, single arm, multicohort, phase 1/2 trial in patients with B-cell malignancies. The CLL cohort included 317 patients, who had previously been treated with at least two prior lines of therapy including a BTKi. The ORR was noted to be similar among patients with WT-BTK with an ORR of 66% and those with C481-mutant with ORR 71% [6]. Regarding the MCL cohort, which included 90 patients with R/R MCL, the ORR was 57.8%, including 20% CR. Pirtobrutinib was well tolerated with low rates of discontinuation (3%) due to treatment-related adverse events [74]. The results of the BRUIN study lead to the accelerated approval of Pirtobrutinib in 2023 for R/R MCL after at least two lines of therapy, including a BTKi and for R/R CLL/SLL after at least two lines of therapy, including a BTKi and a BCL2 inhibitor.

Besides Pirtobrutinib, other noncovalent BTKis are currently under investigation. Nemtabrutinib is being tested in the BELLWAVE-001 (NCT03930953) phase 1/2 trial for R/R CLL/SLL and other B-cell malignancies. Early results from the CLL cohort showed an ORR of 75% with grade 3/4 adverse events including neutropenia (23.4%), febrile neutropenia (14.9%), and pneumonia (14.9%) [75]. Nemtabrutinib is also being investigated in the upfront setting in the phase 3 randomized trial, BELLWAVE-011, comparing nemtabrutinib vs. Ibrutinib or Acalabrutinib in patients with untreated CLL/SLL [76].

Despite the relative short experience with the noncovalent BTKi Pirtobrutinib, progressions and relapses have been described. A recent genomic analysis of pretreatment samples as well samples obtained at that time of progression in R/R CLL patients treated with Pirtobrutinib, identified on-target BTK mutations and also downstream PLCγ2 mutations, that allowed escape from the BTK inhibition [77]. Specifically, a key set of BTK mutations was identified in 9 out of 55 patients with CLL refractory to Pirtobrutinib including V416L, A428D, M437R, T474I, and L528W [77]. All these mutations are clustered in the kinase domain of BTK and can confer resistance to both covalent and noncovalent BTKi [77]. Enzymatic analysis demonstrated that the V416L, A428D, M437R, and L528W resulted in diminished BTK enzymatic function, while the T474I resulted in enhanced BTK enzymatic function. Notably, the T474I and the L528W have also been described in the context of covalent BTK inhibition, highlighting the fact that common mechanism and resistance can occur regardless of the covalent or non-covalent nature of the BTK inhibition. Besides the BTK mutations, genetic studies revealed that PLCγ2 mutations can also confer resistance to Pirtobrutinib. Two patients with stable disease on Pirtobrutinib had preexisting PLCγ2 mutations, E1139 and D1140E, respectively, whereas one patient with preexisting D1144G mutation who initially had a stable response to Pirtobrutinib, developed Richter transformation to DLBCL after 5 months of treatment. 

Understanding the mechanisms of resistance to the non-covalent BTKi, are important in designing the next-generation of therapies. Potential targeting of the scaffold functions of BTK, could disrupt crucial protein–protein interactions, vital for the survival of the cancer cells and reverse the resistance driven by mutations in the kinase domain of BTK. Moreover, combination therapies involving non-covalent inhibitors with other agents may be able to overcome acquired resistant clones and enhance therapy efficacy [78].

### 2.8. BTK Degraders: The Future of BTK Targeted Therapies

BTK degraders represent an emerging alternative in the context of growing resistance to both covalent and noncovalent BTKi. The BTK degraders are proteolysis-targeting chimeras (PROTACs) bifunctional molecules that can bind to the target protein, BTK, and E3 ubiquitin ligase. This binding induces ubiquitination and subsequent proteasomal degradation of the BTK by effectively reducing its levels and inhibiting its signaling pathway [79]. Several BTK degraders are currently in development and key studies and trials have demonstrated the potential of these compounds to overcome resistance and improve outcomes in patients with relapsed or refractory B-cell malignancies.

NX-2127 is one of the first PROTACS to exhibit pre-clinical and clinical efficacy. To synthesize a BTK degrader, a set of numerous BTK binders linked to the cerebron (CRBN) ligand with flexible polyethylene glycol and alkyl linkers of variable length were evaluated for their ability to selectively degrade BTK [80]. Further optimization of the initial compounds was achieved through medical chemistry, structure-based drug design, and empirical testing in both the laboratory and on animal model, leading to the design of NX-2127, an orally bioavailable BTK degrader [80]. NX-2127 was shown to bind to all classes of drug-resistant BTK mutant proteins including BTK proficient and BTK dead/deficient and inducing a stable complex formation with CRBN-DNA damage-binding protein 1 (DDB1). Additionally, NX-2127 was shown to degrade the BTK drug-resistant mutant proteins with subsequent abrogation of the downstream BCR signaling. As a proof of concept, NX-2127 was evaluated in a phase 1 trial for R/R B-cell malignancies which included 29 patients with CLL/SLL among a total of 47 patients [81]. Among the efficacy-evaluable patients with CLL, there were 9 PRs/PR with rebound lymphocytosis, 11 patients with Stable Disease (SD) and 4 with PD. Notably, rapid and robust BTK degradation was observed in all patients regardless of the tumor type. NX-5948 is another BTK degrader, which in contrast to the NX-2127 that degrades both BTK and IKZF3, it selectively degrades BTK. NX-5948 can induce degradation of wild-type and mutant forms of BTK in B-cells at the sub-nanomolar level. NX-5948 is currently being tested in a phase 1a trial (NCT05131022) and preliminary data from 14 patients with R/R CLL or NHL show well tolerability and no discontinuation due to adverse events. All patients had a rapid and sustained reduction in BTK protein due to degradation, regardless of tumor type, drug dose or baseline BTK level [82].

BGB-16673 is another novel BTK degrader, which is currently being tested in patients with R/R B-Cell Malignancies. Preliminary data from a phase 1 trial (NCT05006716) of 26 patients, with at least 2 previous lines of treatment, showed substantial reductions in BTK protein levels in peripheral blood and tumor tissue, even at the lowest dose. Among the 18 response-evaluable patients, 5/6 patients with CLL, 2/2 with MZL and 3/4 with WM demonstrated a PR, whereas 1/3 with MCL had a CR [83]. Notably, a recently published case report described a patient with CLL treated with BGB-16673, who was found to have the A428D mutation at the time of disease progression, suggesting that BGB-16673 may interact with BTK at or around A428 and confer a potential mechanism of resistance [84]. We recognize though that the BTK degraders are still under development, and will require more time to understand the patterns of resistance and for the data to mature.

## 3. Conclusions and Future Directions

The introduction of BTKi has revolutionized the treatment of B-cell malignancies, leading to an improvement in survival rates. In this review, we summarized the clinical data of the landmark clinical trials that led to the FDA approval of the covalent and noncovalent BTKi. We reviewed the mechanisms of resistance to the BTKi by focusing on the BTK mutations and described how the new class of BTK degraders can overcome the BTK proficient and BTK deficient/dead mutations.

Currently, studies assessing novel BTK degraders are underway, whereas trials with combinational programs of BTKi and other molecular targeted therapies are aiming to improve the survival rates of B-cell malignancies. Ultimately, genetic interrogation of patient samples will be vital in understanding the patterns of resistance, as we design new therapies to move the field forward.

## Figures and Tables

**Table 1:** Landmark clinical trials of BTKi. A, acalabrutinib; BR, Bendamustine + Rituximab; Clb, chlorambucil; CLL, chronic lymphocytic leukemia; CR, complete response; FL, follicular lymphoma; m, months; IdR, Idelalisib + Rituximab; MCL, mantle cell lymphoma; MZL, marginal zone lymphoma; N, sample size; NA, not applicable; NHL, non-Hodgkin lymphoma; NR, not reported; O, obinutuzumab; ORR, overall response rate; OS, overall survival; mOS, median OS; PR, partial responses; PFS, progression-free survival; mPFS, median PFS; TN, treatment naïve; R/R, relapsed/refractory; mF/U, median follow-up; VGPR, very good partial response; WM, Waldenstrom macroglobulinemia; Z, zanubrutinib; ZO, zanubrutinib + obinutuzumab.

Study	Regimen	Disease	N	ORR (%)	CR (%)	PFS	OS (m)
Phase I Advani et al. [23]	Ibrutinib	B-cell malignancies-R/R	56	60%	14%	NA	NA
Phase II PCYC-1104 [25]	Ibrutinib	MCL-R/R	111	67%	23%	mPFS: 13m	mOS: 22.5m
Phase III RESONATE [27]	Ibrutinib vs. Ofatumumab	CLL-R/R	391	91% (I)	CR/CRi:11% (I)	mPFS: I-44.1m vs. O-8m	mOS: 67.7m (I)
Phase II Treon et al. [28]	Ibrutinib	WM-R/R	63	90.5% (mF/U 24m)	0	69.1% (mF/U 24m)	95.2% (mF/U 24m)
Phase II Treon et al. [29,30]	Ibrutinib	WM-TN	30	100 (mF/U-50m)	Major response (PR + VGPR) 87% (mF/U-50m)	mPFS at 50m: NR,4-y PFS: 76%	100% (mF/U 50m)
Phase II ACE-LY-004 [33,34]	Acalabrutinib	MCL-R/R	124	81% (mF/U-26m)	43% (mF/U-26m)	mPFS: 20m	mOS: NR (mF/U-26m), estimated 24m OS: 72.4%
Phase III RCTELEVATE-TN [35,36,37]	Acalabrutinib alone or with Obinutuzumab vs. Chlorambucil + Obinutuzumab	CLL-TN	535	A+O-96% vs. A-90% vs. O+Clb-83% (mF/U-46.9m)	A+O-31% vs. A-11% vs. O+Clb-13% (mF/U-46.9m)	mPFS at 74.5m: A+O: NR (72m-PFS 78%) vs. A: NR (72m-PFS 62%) vs. O+Clb: 27.8% (72m-PFS 17m)	Estimated 72m-OS: A+O-84% vs. A-76% vs. O+Clb-75%
Phase III RCTASCEND [38,39]	Acalabrutinib vs. Idelalisib + Rituximab or Bendamustine + Rituximab	CLL-R/R	310	A-83% vs. IdR/BR-84%	A-5% vs. IdR/BR-5%	mPFS at 46.5m: A: NR (42m-PFS 62%) vs. IdR: 16.2m (3y-PFS 23%) vs. BR: 18.6m (3y-PFS 5%)	mOS: NR in either arm (mF/U-46.5m) 3y-OS: A-78% vs. IdR/BR-65%
Phase II Song et al. [40]	Zanubrutinib	MCL-R/R	86	83.70%	77.90%	mPFS: 33m, 3y-PFS: 47.6%	3y-OS: 74.8%
Phase I/II BGB-3111-AU-003 [41]	Zanubrutinib	NHL-R/R	53 pt with R/R MZL/FL	MZL-80% FL-36.4%	MZL-20%, FL-18.2%	mPFS at 33.8m: MZL-NR, FL-10.4 m	mOS: NR for both groups (mF/U 33.8m), 3y-OS 83.9%
Phase III ASPEN [42]	Zanubrutinib vs. Ibrutinib	WM-TN + R/R	221	Z-95.1% vs.I-93.9% (at 60m) in Cohort 1	VGPR + CR: Z-36.3% vs. I-25.3% (mF/U-44.4m) in Cohort 1	mPFS at 44.4m: NR for each group; 3y-PFS:Z-78.3% vs. I-69.7%	mOS: NR for both groups (mF/U 44.4m); 3y-OS: Z-87.5% vs. I-85.2%
Phase II MAGNOLIA [43]	Zanubrutinib	MZL-R/R	68	68.20%	25.80%	mPFS: NR at 15.7m, 15m-PFS: 82.5%	mOS: NR (mF/U 15.7m); 15m-OS: 92.9%
Phase III SEQUOIA [44]	Group A: Zanubrutinib Gourp B:Bendamustine + Rituximab Group C: Zanubrutinib (del17p)	CLL-TN	590	Z-94.6% vs. BR-85.3% (mF/U 26.2m)	Z-7% vs. BR-15% (mF/U 26.2m)	2y-PFS: Z-85.5% vs. BR-69.5%	mOS: NR for both groups (mF/U 26.2m);2y-OS: Z-94.3% vs. BR-94.6%
Phase III ALPINE [45]	Zanubrutinib vs. Ibrutinib	CLL-R/R	652	Z-83.5% vs.I-74.2%	NR	2y-PFS: Z-78.4%vs. I-65.9%	mOS: NR for both groups (mF/U 29.6m)
Phase II ROSEWOOD [46]	Zanubrutinib + obinutuzumab vs. obinutuzumab	R/R FL	217	ZO-69% vs.O-46%	ZO-39% vs. O-19%	mPFS: ZO-28 m vs.O-10.4m	2y-OS: ZO:77% vs. O:71%

## Data Availability

Data sharing is not applicable to this article as no new data were created or analyzed in this study.
